# 1,3-Diallyl-5-chloro-1*H*-benzimidazol-2(3*H*)-one

**DOI:** 10.1107/S1600536811003035

**Published:** 2011-01-29

**Authors:** Younes Ouzidan, Y. Kandri Rodi, Natalie Saffon, El Mokhtar Essassi, Seik Weng Ng

**Affiliations:** a Laboratoire de Chimie Organique Appliquée, Faculté des Sciences et Techniques, Université Sidi Mohamed Ben Abdallah, Fés, Morocco; b Service Commun Rayons-X FR2599, Université Paul Sabatier Bâtiment 2R1, 118 route de Narbonne, Toulouse, France; c Laboratoire de Chimie Organique Hétérocyclique, Pôle de Compétences Pharmacochimie, Université Mohammed V-Agdal, BP 1014 Avenue Ibn Batout, Rabat, Morocco; d Department of Chemistry, University of Malaya, 50603 Kuala Lumpur, Malaysia

## Abstract

The benzimidazolone part of the title mol­ecule, C_13_H_13_ClN_2_O, is almost planar (r.m.s. deviation = 0.006 Å) and its mean plane is aligned at dihedral angles of 62.5 (1) and 78.0 (1)° with respect to the mean planes of the allyl substituents.

## Related literature

For the synthesis, see: Vernin *et al.* (1981 ▶).
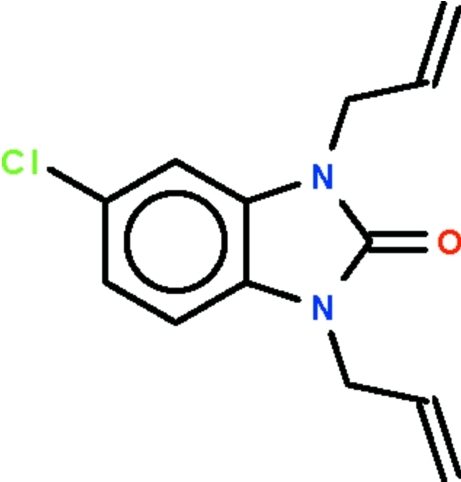


## Experimental

### 

#### Crystal data

C_13_H_13_ClN_2_O
*M* *_r_* = 248.70
Monoclinic, 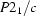

*a* = 7.8831 (1) Å
*b* = 15.2481 (3) Å
*c* = 10.3593 (2) Å
β = 93.056 (1)°
*V* = 1243.44 (4) Å^3^
*Z* = 4
Mo *K*α radiation
μ = 0.29 mm^−1^
*T* = 295 K
0.35 × 0.20 × 0.20 mm

#### Data collection

Bruker APEXII diffractometer
Absorption correction: multi-scan (*SADABS*; Sheldrick, 1997 ▶) *T*_min_ = 0.905, *T*_max_ = 0.944
17723 measured reflections
2858 independent reflections
2230 reflections with *I* > 2σ(*I*)
*R*_int_ = 0.033

#### Refinement

*R*[*F* ^2^ > 2σ(*F* ^2^)] = 0.049
*wR*(*F* ^2^) = 0.156
*S* = 1.03
2858 reflections
154 parameters
H-atom parameters constrained
Δρ_max_ = 0.98 e Å^−3^
Δρ_min_ = −0.34 e Å^−3^

### 

Data collection: *APEX2* (Bruker, 2005 ▶); cell refinement: *SAINT* (Bruker, 2005 ▶); data reduction: *SAINT*; program(s) used to solve structure: *SHELXS97* (Sheldrick, 2008 ▶); program(s) used to refine structure: *SHELXL97* (Sheldrick, 2008 ▶); molecular graphics: *X-SEED* (Barbour, 2001 ▶); software used to prepare material for publication: *publCIF* (Westrip, 2010 ▶).

## Supplementary Material

Crystal structure: contains datablocks global, I. DOI: 10.1107/S1600536811003035/jh2259sup1.cif

Structure factors: contains datablocks I. DOI: 10.1107/S1600536811003035/jh2259Isup2.hkl

Additional supplementary materials:  crystallographic information; 3D view; checkCIF report

## Figures and Tables

**Fig. 1.**
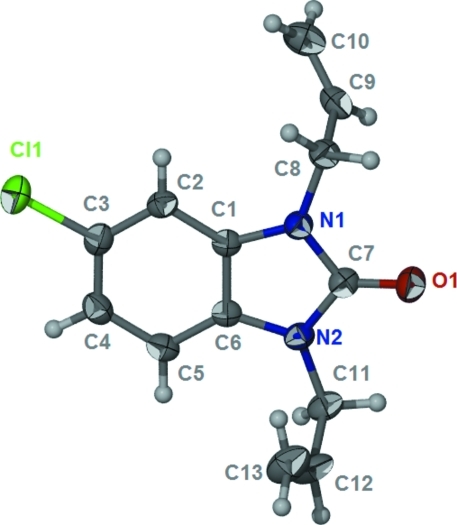
Thermal ellipsoid plot (Barbour, 2001) of C13H13ClN2O at the 50% probability level; hydrogen atoms are drawn as arbitrary radius.

## supplementary crystallographic information

### Comment

Tetraalkylammonium halides are used as phase-transfer catalyst in the synthesis of 1,3-dialkyl-1,2-benzimidazolones, butyltriethylammonium chloride being used in the synthesis of the 1,3-diallyl derivative (Vernin *et al.*, 1981). Thhis compound as well as its derivatives possess pharmalogically important properties. The title chlorine-substitutent compound (Scheme I) was synthesized for evaluation of such properties. The benzimidazolone part of the C_13_H_13_ClN_2_O molecule (Fig. 1) is planar (r.m.s. deviation 0.006 Å); its mean plane is aligned at 62.5 (1) and 78.0 (1) with respect to the mean planes of the allyl substituents.

### Experimental

To 5-chloro-1*H*-benzo[*d*]imidazol-2(3*H*)-one (0.2 g, 1.18 mmol), potassium carbonate (0.4 g, 2.8 mmol), and tetra-*n*-butylammonium bromide (0.08 g, 0.23 mmol) in DMF (15 ml) was added allyl-bromide (0.22 ml, 2.6 mmol). Stirring was continued at room temperature for 6 h. The salts were removed by filtration and the filtrate concentrated under reduced pressure. The residue was separated by chromatography on a column of silica gel with ethyl acetate/hexane (1/2) as eluent. Colorless crystals were isolated when the solvent was allowed to evaporate.

### Refinement

Carbon-bound H-atoms were placed in calculated positions (C—H 0.93–0.97 Å) and were included in the refinement in the riding model approximation, with *U*(H) set to 1.2–1.5*U*(C).

### Crystal data

**Table:**

C_13_H_13_ClN_2_O	*F*(000) = 520
*M**_r_* = 248.70	*D*_x_ = 1.329 Mg m^−^^3^
Monoclinic, *P*2_1_/*c*	Mo *K*α radiation, λ = 0.71073 Å
Hall symbol: -P 2ybc	Cell parameters from 4645 reflections
*a* = 7.8831 (1) Å	θ = 2.4–29.0°
*b* = 15.2481 (3) Å	µ = 0.29 mm^−^^1^
*c* = 10.3593 (2) Å	*T* = 295 K
β = 93.056 (1)°	Block, colourless
*V* = 1243.44 (4) Å^3^	0.35 × 0.20 × 0.20 mm
*Z* = 4	

### Data collection

**Table:**

Bruker APEXII diffractometer	2858 independent reflections
Radiation source: fine-focus sealed tube	2230 reflections with *I* > 2σ(*I*)
graphite	*R*_int_ = 0.033
φ and ω scans	θ_max_ = 27.5°, θ_min_ = 2.4°
Absorption correction: multi-scan (*SADABS*; Sheldrick, 1997)	*h* = −10→10
*T*_min_ = 0.905, *T*_max_ = 0.944	*k* = −19→19
17723 measured reflections	*l* = −12→13

### Refinement

**Table:**

Refinement on *F*^2^	Primary atom site location: structure-invariant direct methods
Least-squares matrix: full	Secondary atom site location: difference Fourier map
*R*[*F*^2^ > 2σ(*F*^2^)] = 0.049	Hydrogen site location: inferred from neighbouring sites
*wR*(*F*^2^) = 0.156	H-atom parameters constrained
*S* = 1.03	*w* = 1/[σ^2^(*F*_o_^2^) + (0.0846*P*)^2^ + 0.6164*P*] where *P* = (*F*_o_^2^ + 2*F*_c_^2^)/3
2858 reflections	(Δ/σ)_max_ = 0.001
154 parameters	Δρ_max_ = 0.98 e Å^−^^3^
0 restraints	Δρ_min_ = −0.34 e Å^−^^3^

### Fractional atomic coordinates and isotropic or equivalent isotropic displacement parameters (Å^2^)

**Table:**

	*x*	*y*	*z*	*U*_iso_*/*U*_eq_	
Cl1	0.25390 (9)	0.66457 (5)	0.19928 (6)	0.0609 (2)	
O1	0.6243 (2)	0.62064 (10)	0.86933 (14)	0.0468 (4)	
N1	0.5616 (2)	0.66732 (10)	0.65711 (15)	0.0341 (4)	
N2	0.4252 (2)	0.55197 (10)	0.72844 (15)	0.0340 (4)	
C1	0.4530 (2)	0.63903 (12)	0.55655 (17)	0.0304 (4)	
C2	0.4221 (2)	0.67167 (12)	0.43253 (18)	0.0346 (4)	
H2	0.4781	0.7208	0.4029	0.042*	
C3	0.3021 (3)	0.62631 (14)	0.35525 (19)	0.0388 (5)	
C4	0.2168 (3)	0.55288 (14)	0.3964 (2)	0.0407 (5)	
H4	0.1391	0.5244	0.3403	0.049*	
C5	0.2476 (2)	0.52151 (13)	0.5227 (2)	0.0371 (4)	
H5	0.1904	0.4728	0.5526	0.045*	
C6	0.3663 (2)	0.56580 (12)	0.60094 (17)	0.0307 (4)	
C7	0.5461 (3)	0.61349 (12)	0.76399 (18)	0.0348 (4)	
C8	0.6889 (3)	0.73629 (13)	0.6498 (2)	0.0385 (5)	
H8A	0.7127	0.7607	0.7353	0.046*	
H8B	0.6442	0.7829	0.5941	0.046*	
C9	0.8507 (3)	0.70238 (14)	0.5983 (2)	0.0438 (5)	
H9	0.9055	0.6560	0.6414	0.053*	
C10	0.9186 (3)	0.73440 (16)	0.4965 (3)	0.0548 (6)	
H10A	0.8664	0.7808	0.4516	0.066*	
H10B	1.0192	0.7109	0.4687	0.066*	
C11	0.3710 (3)	0.48204 (13)	0.8125 (2)	0.0410 (5)	
H11A	0.4544	0.4757	0.8842	0.049*	
H11B	0.3681	0.4274	0.7645	0.049*	
C12	0.2008 (3)	0.49716 (17)	0.8649 (2)	0.0561 (7)	
H12	0.1536	0.4507	0.9088	0.067*	
C13	0.1120 (4)	0.5692 (2)	0.8550 (3)	0.0647 (8)	
H13A	0.1540	0.6174	0.8120	0.078*	
H13B	0.0066	0.5723	0.8911	0.078*	

### Atomic displacement parameters (Å^2^)

**Table:**

	*U*^11^	*U*^22^	*U*^33^	*U*^12^	*U*^13^	*U*^23^
Cl1	0.0596 (4)	0.0792 (5)	0.0427 (3)	−0.0024 (3)	−0.0096 (3)	0.0139 (3)
O1	0.0592 (10)	0.0442 (8)	0.0362 (7)	−0.0043 (7)	−0.0055 (7)	0.0002 (6)
N1	0.0399 (9)	0.0296 (8)	0.0326 (8)	−0.0039 (6)	0.0018 (7)	−0.0010 (6)
N2	0.0393 (9)	0.0291 (8)	0.0339 (8)	−0.0015 (6)	0.0054 (6)	0.0019 (6)
C1	0.0300 (9)	0.0290 (8)	0.0329 (9)	0.0024 (7)	0.0062 (7)	−0.0024 (7)
C2	0.0335 (10)	0.0357 (10)	0.0352 (9)	0.0027 (7)	0.0070 (8)	0.0034 (7)
C3	0.0355 (10)	0.0477 (11)	0.0335 (9)	0.0087 (8)	0.0041 (8)	0.0005 (8)
C4	0.0320 (10)	0.0473 (11)	0.0427 (11)	−0.0002 (8)	0.0013 (8)	−0.0081 (9)
C5	0.0332 (10)	0.0350 (9)	0.0437 (10)	−0.0031 (8)	0.0069 (8)	−0.0039 (8)
C6	0.0303 (9)	0.0288 (8)	0.0336 (9)	0.0028 (7)	0.0071 (7)	−0.0023 (7)
C7	0.0406 (10)	0.0288 (9)	0.0351 (9)	0.0020 (8)	0.0043 (8)	−0.0014 (7)
C8	0.0465 (12)	0.0300 (9)	0.0391 (10)	−0.0081 (8)	0.0024 (8)	−0.0036 (8)
C9	0.0348 (11)	0.0325 (10)	0.0627 (13)	−0.0023 (8)	−0.0097 (9)	0.0045 (9)
C10	0.0429 (13)	0.0474 (12)	0.0750 (17)	−0.0005 (10)	0.0119 (11)	−0.0051 (12)
C11	0.0500 (12)	0.0313 (10)	0.0424 (11)	0.0032 (8)	0.0094 (9)	0.0089 (8)
C12	0.0663 (16)	0.0553 (14)	0.0492 (13)	0.0046 (12)	0.0263 (12)	0.0163 (10)
C13	0.0656 (17)	0.0776 (18)	0.0535 (14)	0.0202 (14)	0.0259 (13)	0.0098 (13)

### Geometric parameters (Å, °)

**Table:**

Cl1—C3	1.741 (2)	C5—H5	0.9300
O1—C7	1.229 (2)	C8—C9	1.501 (3)
N1—C1	1.382 (2)	C8—H8A	0.9700
N1—C7	1.389 (2)	C8—H8B	0.9700
N1—C8	1.459 (2)	C9—C10	1.303 (3)
N2—C7	1.373 (3)	C9—H9	0.9300
N2—C6	1.393 (2)	C10—H10A	0.9300
N2—C11	1.455 (2)	C10—H10B	0.9300
C1—C2	1.387 (3)	C11—C12	1.492 (3)
C1—C6	1.400 (3)	C11—H11A	0.9700
C2—C3	1.390 (3)	C11—H11B	0.9700
C2—H2	0.9300	C12—C13	1.303 (4)
C3—C4	1.385 (3)	C12—H12	0.9300
C4—C5	1.402 (3)	C13—H13A	0.9300
C4—H4	0.9300	C13—H13B	0.9300
C5—C6	1.381 (3)		
			
C1—N1—C7	109.83 (16)	N2—C7—N1	106.21 (16)
C1—N1—C8	125.89 (16)	N1—C8—C9	111.75 (16)
C7—N1—C8	123.90 (17)	N1—C8—H8A	109.3
C7—N2—C6	110.02 (15)	C9—C8—H8A	109.3
C7—N2—C11	124.17 (17)	N1—C8—H8B	109.3
C6—N2—C11	125.80 (17)	C9—C8—H8B	109.3
N1—C1—C2	131.12 (17)	H8A—C8—H8B	107.9
N1—C1—C6	107.17 (16)	C10—C9—C8	123.5 (2)
C2—C1—C6	121.71 (18)	C10—C9—H9	118.2
C3—C2—C1	115.81 (18)	C8—C9—H9	118.2
C3—C2—H2	122.1	C9—C10—H10A	120.0
C1—C2—H2	122.1	C9—C10—H10B	120.0
C4—C3—C2	123.46 (19)	H10A—C10—H10B	120.0
C4—C3—Cl1	118.11 (17)	N2—C11—C12	113.75 (17)
C2—C3—Cl1	118.43 (16)	N2—C11—H11A	108.8
C3—C4—C5	120.04 (19)	C12—C11—H11A	108.8
C3—C4—H4	120.0	N2—C11—H11B	108.8
C5—C4—H4	120.0	C12—C11—H11B	108.8
C6—C5—C4	117.31 (18)	H11A—C11—H11B	107.7
C6—C5—H5	121.3	C13—C12—C11	126.3 (2)
C4—C5—H5	121.3	C13—C12—H12	116.9
C5—C6—N2	131.58 (17)	C11—C12—H12	116.9
C5—C6—C1	121.65 (18)	C12—C13—H13A	120.0
N2—C6—C1	106.76 (16)	C12—C13—H13B	120.0
O1—C7—N2	127.35 (18)	H13A—C13—H13B	120.0
O1—C7—N1	126.44 (18)		
			
C7—N1—C1—C2	179.23 (19)	C2—C1—C6—C5	1.1 (3)
C8—N1—C1—C2	−7.7 (3)	N1—C1—C6—N2	0.17 (19)
C7—N1—C1—C6	0.2 (2)	C2—C1—C6—N2	−178.95 (16)
C8—N1—C1—C6	173.33 (17)	C6—N2—C7—O1	179.96 (19)
N1—C1—C2—C3	−179.86 (18)	C11—N2—C7—O1	−1.2 (3)
C6—C1—C2—C3	−1.0 (3)	C6—N2—C7—N1	0.6 (2)
C1—C2—C3—C4	−0.1 (3)	C11—N2—C7—N1	179.50 (16)
C1—C2—C3—Cl1	178.90 (14)	C1—N1—C7—O1	−179.86 (19)
C2—C3—C4—C5	1.1 (3)	C8—N1—C7—O1	6.9 (3)
Cl1—C3—C4—C5	−177.91 (15)	C1—N1—C7—N2	−0.5 (2)
C3—C4—C5—C6	−1.0 (3)	C8—N1—C7—N2	−173.81 (16)
C4—C5—C6—N2	179.97 (18)	C1—N1—C8—C9	−83.2 (2)
C4—C5—C6—C1	−0.1 (3)	C7—N1—C8—C9	88.9 (2)
C7—N2—C6—C5	179.44 (19)	N1—C8—C9—C10	122.9 (2)
C11—N2—C6—C5	0.6 (3)	C7—N2—C11—C12	104.9 (2)
C7—N2—C6—C1	−0.5 (2)	C6—N2—C11—C12	−76.4 (3)
C11—N2—C6—C1	−179.34 (17)	N2—C11—C12—C13	−9.0 (4)
N1—C1—C6—C5	−179.79 (17)		
